# Management of Hepatocellular Carcinoma with Advanced Portal Vein Tumor Thrombus: Current Evidence and Future Perspectives—A Narrative Review

**DOI:** 10.3390/cancers18152380

**Published:** 2026-07-23

**Authors:** Hiroyuki Kato, Zenichi Morise, Yutaro Kato, Hidetoshi Katsuno, Masahiro Ito, Akihiko Horiguchi

**Affiliations:** 1 Department of Gastroenterological Surgery, Fujita Health University School of Medicine, Bantane Hospital, 3-6-10 Otobashi, Nakagawa-ku, Nagoya 454-8509, Aichi, Japan; zmorise@fujita-hu.ac.jp (Z.M.); masito@fujita-hu.ac.jp (M.I.); akihori@fujita-hu.ac.jp (A.H.); 2 Department of Surgery, Fujita Health University School of Medicine, Okazaki Medical Center, 2-6-1 Harisaki-nishi, Okazaki 444-0829, Aichi, Japan

**Keywords:** hepatocellular carcinoma, portal vein tumor thrombus, PVTT, conversion surgery, hepatectomy, immune checkpoint inhibitor, multidisciplinary treatment

## Abstract

Portal vein tumor thrombus (PVTT) is one of the most critical adverse prognostic factors in hepatocellular carcinoma (HCC) and is associated with rapid intrahepatic progression, deterioration of liver function, and extremely poor survival. Although Western guidelines generally recommend systemic therapy for HCC with PVTT, increasing evidence from Asian countries has demonstrated that selected patients may benefit from aggressive multidisciplinary treatment strategies including surgical resection, hepatic arterial infusion chemotherapy (HAIC), transarterial chemoembolization (TACE), radiotherapy, and immune checkpoint inhibitor (ICI)-based systemic therapy. Historically, surgical resection has shown survival benefit mainly in Vp1–Vp3 disease, whereas the role of surgery in Vp4 disease remains controversial because prognosis is strongly influenced by tumor biology and liver functional reserve. Recent advances in ICI- and tyrosine kinase inhibitor (TKI)-based therapies have further expanded the feasibility of conversion surgery for initially unresectable disease. Tumor markers, including AFP and DCP, treatment response, and liver function may provide complementary prognostic information in addition to anatomical PVTT extent when determining prognosis and treatment strategy. In this review, we summarize the historical evolution and current evidence regarding surgical resection, perioperative therapy, systemic therapy, and conversion surgery for HCC with PVTT, particularly focusing on advanced Vp3/Vp4 disease. We also discuss the evolving concept of multidisciplinary treatment integration in the immunotherapy era and propose an evidence-based treatment algorithm for clinical practice.

## Simple Summary

Portal vein tumor thrombus is a serious complication of liver cancer that is associated with rapid disease progression and poor survival. Although drug therapy is generally recommended, selected patients may benefit from surgery or combinations of surgery, radiotherapy, catheter-based treatment, and modern immunotherapy. This review summarizes how treatment has evolved, with particular attention to advanced tumor thrombus involving major branches or the main portal vein. We examine the factors that may help identify patients who could benefit from surgery, the growing role of treatment before surgery and conversion surgery, and important differences between Asian and Western practice. We also propose a practical multidisciplinary treatment framework. The review may help clinicians balance tumor extent, liver function, treatment response, and patient condition when selecting individualized treatment, while recognizing that stronger prospective evidence is still needed.

## 1. Background

The presence of portal vein tumor thrombus (PVTT) in hepatocellular carcinoma (HCC) is widely recognized as one of the most critical determinants of tumor progression and prognosis [1]. According to the Japanese classification of liver cancer, VP3 (invasion of the first-order portal branches) and VP4 (involvement of the main trunk or contralateral branch) represent advanced disease stages and are associated with extremely poor outcomes [2,3]. In particular, VP4 disease reflects not only severe impairment of portal blood flow but also aggressive tumor biology, and is often regarded clinically as having a behavior similar to systemic disease [4]. Consequently, local therapies have traditionally been considered of limited value, and systemic therapy has been the mainstay of treatment for these patients.

HCC is characterized by a high propensity for invasion and dissemination via the portal venous system. Despite the presence of two distinct venous systems in the liver—the hepatic veins and the portal veins—the mechanisms underlying the preferential spread of tumor cells through the portal circulation remain incompletely understood. Mitsunobu et al. [5] reported that, following direct injection of contrast medium into resected tumor specimens, drainage occurred exclusively through the portal venous system in 74% of cases, suggesting that portal-dominant hemodynamics may contribute to the formation of PVTT. Recent studies have suggested that PVTT formation is associated with complex molecular mechanisms, including epithelial–mesenchymal transition (EMT), angiogenesis, and cancer stem cell properties. Activation of EMT-related pathways promotes tumor invasion and vascular infiltration [6], while angiogenic factors such as VEGF contribute to tumor vascularization and portal vein invasion [7]. In addition, cancer stem cell-like characteristics may be involved in aggressive tumor behavior and resistance to therapy [8]. Understanding these biological mechanisms may contribute to the development of systemic therapies and multidisciplinary strategies for HCC with PVTT.

This review focuses on advanced HCC with PVTT, particularly Vp3/Vp4 disease, and summarizes the evolution of surgical, locoregional, and systemic treatments. We integrate recent evidence on immune checkpoint inhibitor-based therapy, conversion surgery, and biological resectability, highlight differences between Western and Asian treatment paradigms, and propose an evidence-informed framework for multidisciplinary decision-making.

## 2. Literature Search Strategy

PubMed and Scopus were searched for English-language articles published between January 2016 and July 2026 using the following terms: (“hepatocellular carcinoma” OR “HCC”) AND (“portal vein tumor thrombus” OR “PVTT” OR “macrovascular invasion” OR “vascular invasion”) AND (“hepatectomy” OR “surgical resection” OR “conversion surgery” OR “immune checkpoint inhibitor” OR “systemic therapy” OR “HAIC” OR “TACE” OR “radiotherapy” OR “guideline” OR “borderline resectable” OR “prognostic factor” OR “meta-analysis”).

**Figure 1:**
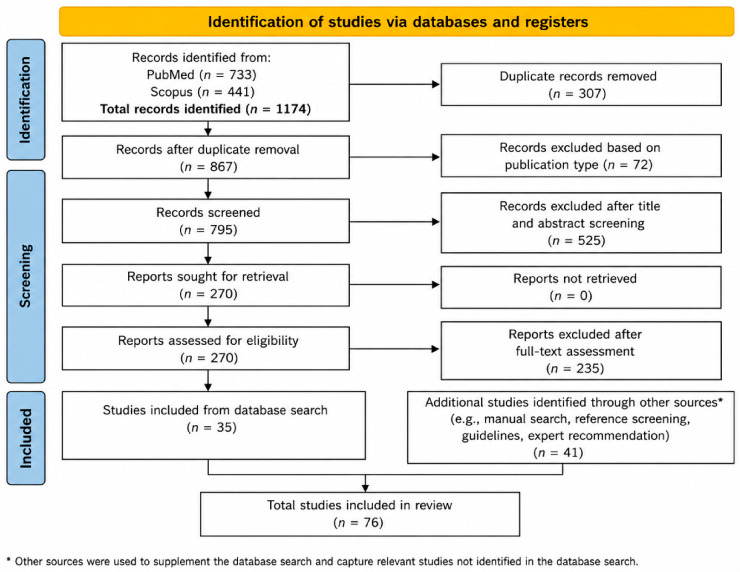
PRISMA-style flow diagram of the literature identification and selection process.

The search identified 1174 records (PubMed, *n* = 733; Scopus, *n* = 441). After removing 307 duplicates and 72 records with ineligible publication types, 795 records underwent title and abstract screening. Eligible study designs included randomized controlled trials, systematic reviews, meta-analyses, propensity score-matched studies, and retrospective case–control or cohort studies. Case series and case reports were included only when they described pioneering treatment strategies. Two reviewers (HK and ZM) independently selected English-language studies primarily evaluating treatment outcomes for HCC with major PVTT, principally Vp3/Vp4. Of the 795 records screened, 270 full-text reports were assessed for eligibility, of which 235 were excluded, leaving 35 reports included from the database search. Disagreements between the reviewers were resolved through discussion. An additional 41 references were identified through citation searching, targeted searches for historical studies and guidelines, and supplementary searches prompted by peer-review comments, resulting in 76 references overall (Figure 1).

To clarify the applicability of the available evidence to advanced PVTT, the included studies were classified according to their study populations. Studies specifically evaluating patients with Vp3/Vp4 or major PVTT included references [3,4,9,10,11,12,13]. Studies involving mixed or unstratified PVTT populations, as well as broader HCC cohorts that included patients with PVTT or reported PVTT-related findings, included references [1,2,14,15,16,17,18,19,20,21,22,23,24,25,26,27,28,29,30,31,32,33,34,35,36,37,38,39,40,41,42,43,44,45,46,47,48,49,50,51,52,53,54]. Studies evaluating macrovascular or vascular invasion without separately reporting outcomes for PVTT included references [55,56,57,58,59,60,61]. Broader HCC studies without an identifiable PVTT population included references [62,63,64,65,66]. Findings from the latter three categories were regarded as indirect evidence for Vp3/Vp4 disease unless Vp3/Vp4-specific subgroup results were available. Mechanistic studies, reviews, consensus statements, surveys, and clinical guidelines were used as contextual references [5,6,7,8,67,68,69,70,71,72,73,74,75,76].

## 3. Patient Selection for Hepatic Resection in Patients with HCC and PVTT

Patient selection for hepatic resection in patients with HCC and PVTT should not be based solely on technical resectability. The Chinese expert consensus recommends surgery primarily for patients with Child–Pugh class A liver function, an ECOG performance status of 0–1, and type I/II PVTT; selected patients with type III PVTT may also be considered for surgery, whereas Child–Pugh class C liver dysfunction, massive ascites, variceal gastrointestinal bleeding, or hepatic encephalopathy generally favor supportive or nonsurgical management [67]. More broadly, contemporary HCC surgical guidance emphasizes multidisciplinary assessment incorporating liver function, portal hypertension, tumor burden, and anticipated perioperative risk [55]. In a retrospective study of 161 patients who underwent hepatectomy for HCC with PVTT, Kim et al. reported 1-, 3-, and 5-year overall survival rates of 65.0%, 38.4%, and 36.0%, respectively [14]. The absence of esophageal varices, a maximum tumor diameter of <5 cm, tumor confinement to a single hepatic lobe, and anatomical resection were independently associated with more favorable overall survival, while R0 resection and the absence of microvascular invasion were associated with improved recurrence-free survival. These findings suggest that clinically significant portal hypertension and extensive intrahepatic tumor burden may reduce the expected benefit of surgery. Among 100 patients undergoing hepatectomy for Vp3/Vp4 PVTT, Komatsu et al. reported a median overall survival of 14.5 months and 1- and 3-year overall survival rates of 59.6% and 16.8%, respectively, highlighting that anatomical resectability does not necessarily translate into durable oncological benefit [3]. The presence of extrahepatic disease, poor performance status, major comorbidities, or clinically relevant frailty should therefore weigh against extensive hepatectomy because these factors may limit postoperative recovery, delay subsequent systemic treatment, and compromise independent living. Although PVTT-specific evidence regarding frailty and patient-reported outcomes remains limited, a study of 128 patients undergoing resection of primary or metastatic hepatic malignancies showed that preoperative health-related quality of life was independently associated with survival; quality of life initially deteriorated after surgery but subsequently returned toward baseline [62]. Final treatment decisions should therefore be made through multidisciplinary review and shared decision-making that explicitly considers anticipated survival benefit, the risk of post-hepatectomy liver failure and other major complications, postoperative quality of life, and the fully informed patient’s preferences.

## 4. Prognostic Biomarkers After Resection for HCC with PVTT

As for the prognostic factors after resection for HCC with PVTT, multiple studies have identified preoperative factors predictive of poor outcomes following hepatectomy for HCC with PVTT (Table 1). Elevated AFP is the most consistently reported factor: Roayaie et al. [56] identified AFP > 30 ng/mL as an independent predictor (HR 2.07), Chen et al. [15] confirmed AFP > 400 ng/mL as a determinant of actual long-term survival, and Huo et al. [16] demonstrated that elevated AFP predicted both early death (≤3 months) and long-term mortality. Tumor size is another established factor; Kim et al. [14] reported tumor size ≥ 5 cm as an independent predictor of poor OS, and the MRI-based model by Zhang et al. [17] identified the sum of the two largest tumor diameters as the strongest predictor (HR 3.05). Hepatic functional reserve critically influences outcomes. Huo et al. [16] identified elevated total bilirubin and radiologic ascites as predictors of early postoperative death, while clinically significant portal hypertension was associated with poor long-term survival. Kim et al. [14] reported esophageal varices as an independent poor prognostic factor, and the ASCO GI 2023 Western cohort demonstrated an MST of 16.0 months for Child-Pugh A versus 3.6 months for Child-Pugh B [18]. Several preoperative scoring systems integrate these factors. The EHBH-PVTT score (total bilirubin, AFP, tumor diameter, satellite lesions) stratified patients into distinct prognostic groups (MST 17.0 vs. 7.9 months; *p* < 0.001) [19]. The ADV score (AFP × DCP × tumor volume) by Hwang et al. demonstrated superior prognostic accuracy and notably found that Vp classification itself was not a significant prognostic factor when the ADV score was accounted for [20]. EHBH-PVTT may be useful for initial prognostic stratification in advanced PVTT because it incorporates PVTT extent and liver function, whereas the ADV score provides complementary information regarding tumor biology and recurrence risk.

Miura et al. (2026) further validated that significant tumor marker elevation (AFP > 500 ng/mL or DCP > 1000 mAU/mL) provides independent prognostic information beyond anatomical resectability criteria [24]. These findings collectively indicate that prognosis after hepatectomy for HCC with PVTT is determined primarily by tumor biological aggressiveness (AFP, DCP, tumor size) and hepatic functional reserve, rather than by the anatomical extent of PVTT alone. In addition to conventional tumor-related factors, emerging prognostic markers include the neutrophil-to-lymphocyte ratio [58], sarcopenia [63], ALBI grade [64], radiomics-based imaging biomarkers [25], and circulating tumor DNA [65] which have also recently attracted attention for predicting prognosis and treatment response in patients with advanced HCC. Immune-related genomic biomarkers, such as chromosome 11q13 amplification, may also predict resistance to PD-1 blockade, although evidence specific to HCC with PVTT remains limited [26]. Available retrospective studies suggest that carefully selected patients with Vp1–Vp3 disease may achieve superior survival with resection compared with non-surgical therapies; however, the evidence is subject to substantial selection bias and lacks confirmation from randomized trials. Therefore, surgical resection should be regarded as a potential treatment option within a multidisciplinary framework rather than an established standard therapy, particularly for advanced Vp3/Vp4 disease. Even in technically resectable Vp3/Vp4 disease, patients with high tumor marker levels or compromised liver function may derive limited benefit from surgical resection alone.

## 5. Role of Surgery According to the Extent of PVTT

Surgical resection for hepatocellular carcinoma (HCC) with portal vein tumor thrombus (PVTT) has long been considered contraindicated in Western guidelines, where PVTT categorizes patients as Barcelona Clinic Liver Cancer (BCLC) stage C with systemic therapy as the sole recommendation. In contrast, selective surgical resection has been actively pursued in Japan and China since the late 1990s [9,27,68,69]. One of the earliest reports was by Yamaoka et al. (1992), who performed hepatectomy with direct thrombectomy of the main portal vein in 29 patients; although initially undertaken as an emergency procedure to prevent variceal rupture, the 1-year and 3-year survival rates were 52.2% and 11.6%, respectively, significantly superior to non-surgical management [9]. Subsequently, Minagawa and Makuuchi (2001), analyzing 45 patients treated between 1989 and 1998, demonstrated that preoperative TACE followed by hepatectomy yielded 1-year and 5-year survival rates of 82% and 42%, with hepatectomy being the only independent favorable prognostic factor on multivariate analysis [27]. These pioneering studies established the foundation for selective surgical resection of HCC with PVTT in Japan. The landmark Japanese nationwide survey by Kokudo et al. (2016) [21] analyzed 6474 patients with HCC and PVTT. In Child-Pugh A patients, the median survival time (MST) was 2.87 years in the resection group versus 1.10 years in the non-resection group (*p* = 0.001), with a 0.88-year advantage persisting after propensity score matching. However, the survival benefit was not statistically significant for Vp4 disease. The 90-day postoperative mortality was 3.7%. In a nationwide Japanese cohort as shown in Table 2, median overall survival decreased stepwise according to the extent of portal vein invasion, with MSTs of 80.6 months in Vp1, 36.9 months in Vp2, 24.8 months in Vp3, and 14.1 months in Vp4, respectively, while even Vp4 patients demonstrated a 5-year survival rate of approximately 19% [70].

For Vp3/Vp4 disease specifically, Komatsu et al. (2022) [3] reported an MST of 14.5 months in 100 patients, with no significant difference between Vp3 and Vp4 (16.1 vs. 14.3 months; *p* = 0.71) or between R0/R1 and R2 resection, suggesting that tumor biology predominantly determines prognosis at this level. In summary, surgical resection may demonstrate a survival benefit for Vp1–Vp3 disease, but the benefit for Vp4 remains limited, underscoring the importance of patient selection.

However, the evidence supporting hepatic resection for Vp3/Vp4 PVTT should be interpreted cautiously. In a Japanese bi-institutional propensity-matched study, hepatectomy was associated with longer survival than sorafenib; however, only 36 patients were included in each matched group, and residual treatment-selection bias could not be excluded [10]. A systematic review of seven comparative studies involving 4810 patients similarly reported better survival after resection, but all included comparisons were nonrandomized and involved selected patients with resectable disease [28]. Moreover, Western evidence directly applicable to Vp3/Vp4 disease remains limited. In the Italian multicenter study by Famularo et al., patients with main portal trunk invasion were excluded and outcomes were not stratified by PVTT grade; therefore, the study does not provide direct evidence for hepatectomy in Vp4 disease [59]. Accordingly, hepatic resection should be considered only for highly selected patients with preserved liver function, no extrahepatic disease, and technically resectable tumor extension.

## 6. Conversion Surgery

### 6.1. Preoperative Treatment and Surgical Outcomes

The combination of ICIs and tyrosine kinase inhibitors (TKIs) has enabled conversion surgery for initially unresectable HCC with PVTT. A meta-analysis by Rajagopal et al. (2026; 7 trials, 621 patients) demonstrated that neoadjuvant therapy plus resection significantly improved OS (HR 0.48) and RFS (HR 0.40) compared with resection alone for HCC with PVTT [29]. The only RCT in this setting, by Wei et al. (2019, JCO), showed that neoadjuvant 3D-CRT plus resection significantly improved OS compared with resection alone (24-month OS: 27.4% vs. 9.4%; *p* < 0.001) [30]. Hu et al. (2023) also demonstrated that neoadjuvant HAIC plus resection improved 5-year OS to 66.4% versus 37.2% with resection alone, though the benefit was confined to HAIC responders [31].

A meta-analysis of conversion surgery (Xu et al., 2024 [32]; 38 studies, 4042 patients) reported conversion rates of 8% with TKI alone, 28% with TKI plus anti-PD-1, and 33–35% with TKI plus anti-PD-1 plus locoregional therapy. Conversion surgery significantly improved OS (HR 0.45) and PFS (HR 0.49) [32]. Zhang et al. (2026) reported a conversion resection rate of 59.2% with lenvatinib plus TACE plus anti-PD-1 (LEN-TAP) versus 18.3% with TACE alone, with markedly improved OS and EFS [33]. For Vp4 PVTT specifically, the addition of a PD-1 inhibitor to HAIC plus a TKI was associated with higher overall and PVTT-specific response rates and longer PFS and OS than HAIC plus a TKI alone, likely because HAIC more effectively controls intraluminal tumor thrombus within the portal vein [11].

However, the necessity of conversion surgery in all responders remains debated. Wang et al. (2024) found no significant OS or PFS difference between surgical and non-surgical groups among patients who achieved successful conversion, particularly those with complete response or Cheng type III–IV PVTT [34]. However, this study was limited by its retrospective design, small sample size, and lack of stratification according to pathological response, including MPR and pCR.

The NeoHCC Consortium (D’Alessio et al., 2024 [66], Lancet Oncology) reported an MPR rate of 32% and pCR rate of 18% after neoadjuvant ICI, with MPR strongly associated with improved RFS (HR 0.26; *p* = 0.0024). Notably, 30% of patients achieving MPR did not show radiological response, highlighting the discordance between imaging and pathological assessment [66]. Wang et al. (2026) suggested that 4–6 months of postoperative TKI plus ICI represents the optimal adjuvant duration after conversion surgery [35].

Although conversion surgery after effective systemic therapy has shown promising outcomes, current evidence should be interpreted cautiously. Since the IMbrave150 trial demonstrated that atezolizumab plus bevacizumab significantly improved survival compared with sorafenib in patients with unresectable HCC, the incremental survival benefit attributable to surgery after a favorable response to modern systemic therapy remains uncertain [36]. Thus, patients eligible for conversion surgery represent a highly selected population with favorable tumor biology and good treatment response, creating substantial selection bias. Therefore, improved survival after conversion surgery may partly reflect underlying tumor sensitivity to systemic therapy rather than the effect of surgery itself. Prospective randomized trials comparing conversion surgery with continued systemic therapy are required to define the true survival benefit. The principal studies on neoadjuvant, conversion, and perioperative therapy are summarized in Table 3.

### 6.2. Resectability and Conversion Criteria

The Japan Liver Cancer Study Group (JLCSG) and the Japanese Society of Hepato-Biliary-Pancreatic Surgery (JSHBPS) proposed oncological resectability criteria in 2023, classifying HCC into three categories: Resectable (R), Borderline Resectable 1 (BR1; one adverse factor: ≥4 tumors, tumor size > 5 cm, or macrovascular invasion), and Borderline Resectable 2 (BR2; two or more adverse factors). A nationwide questionnaire survey by Akahoshi et al. (2024) revealed that 90.9% of Japanese board-certified hepatobiliary surgeons considered Vp1 resectable, 70.7% for Vp2, but only 39.0% for Vp3 and 8.0% for Vp4, reflecting the lack of consensus on surgical indications for advanced PVTT [71].

Shindoh et al. (2025) demonstrated in a multicenter study (BR-HCC, *n* = 1509) that preoperative systemic therapy significantly improved DSS (HR 0.41) and RFS (HR 0.80) in BR1 patients, whereas no significant benefit was observed in BR2 patients [39]. Magyar et al. (2025) positioned patients with macrovascular invasion as generally “borderline resectable” in a comprehensive review published in Lancet Gastroenterology Hepatology [72].

In addition to these oncological criteria, biological factors including AFP, DCP, and the ADV score may provide complementary information regarding tumor aggressiveness and help guide treatment selection, including upfront surgery versus initial systemic therapy followed by response-based reassessment and biological refinement. Aso et al. (2026) identified a “biological-BR” subgroup within the R category, defined by AFP ≥ 400 ng/mL and DCP ≥ 400 mAU/mL, which exhibited an MVI rate of 42.3% and outcomes comparable to BR1 [40]. Miura et al. (2026) similarly reported that tumor marker-high status (AFP > 500 ng/mL or DCP > 1000 mAU/mL) was an independent prognostic factor regardless of resectability classification [24]. Komatsu et al. (2026) further demonstrated that macrovascular invasion was the most impactful factor among BR criteria (BR1: MST 34.2 vs. 63.4 months, *p* = 0.04; BR2: MST 14.4 vs. 20.9 months, *p* = 0.004), while in systemic chemotherapy responders, the correlation between tumor burden and prognosis was abolished (25.4 vs. 24.5 months, *p* = 0.502), supporting the rationale for conversion therapy [41]. Standardized criteria for conversion surgery candidacy have not been established. Xu et al. (2022) proposed the following prerequisites: ECOG PS 0–1, Child-Pugh A, tumor confined to one lobe, absence of contralateral portal vein invasion, and IVC tumor thrombus not extending to the atrium [42]. Treatment response assessment by mRECIST (PR or better) and tumor marker kinetics (AFP > 50% decline or PIVKA-II > 75% decline) have been associated with successful conversion to resection. The NCCN guidelines (2026) state that patients with initially unresectable disease who respond to therapy may be considered for surgery, with multidisciplinary team discussion recommended for determining the optimal timing of surgery after systemic therapy.

### 6.3. Postoperative Management and Adjuvant Therapy

As mentioned above, postoperative recurrence rates in HCC with PVTT exceed 70% at 5 years, making effective adjuvant therapy an urgent priority. A summary of studies on adjuvant therapy after surgical resection for HCC with or at risk of PVTT is shown in Table 4.

Peng et al. (2024) [43] reported the first phase 3 RCT of adjuvant therapy specifically for PVTT. In 158 patients with Cheng type I–III PVTT, adjuvant TACE plus sorafenib versus sorafenib alone demonstrated improved median RFS (16.8 vs. 12.6 months; HR 0.57; *p* = 0.002) and OS (30.4 vs. 22.5 months; HR 0.57; *p* = 0.02) without additional toxicity [43]. A Bayesian network meta-analysis by Sun et al. (2023) of 14 trials (1927 patients) found all adjuvant therapies superior to resection alone, with radiotherapy being the most effective (OS: HR 0.38; RFS: HR 0.27) [44]. Regarding immune checkpoint inhibitor (ICI)-based adjuvant therapy, the IMbrave050 trial initially showed improved RFS with atezolizumab plus bevacizumab (HR 0.72; *p* = 0.012), but the benefit was not sustained at longer follow-up (HR 0.90; 95% CI 0.72–1.12) [45,46]. Based on these data, the AASLD 2025 Critical Update consequently does not recommend ICI-based adjuvant therapy after resection [45,73].

In contrast, the CARES-009 trial (Wang et al., 2025 [38]) demonstrated that a perioperative approach—camrelizumab plus rivoceranib given both before and after surgery—significantly improved event-free survival (EFS) in resectable HCC at intermediate-to-high recurrence risk, including patients with vascular invasion. This suggests that combining neoadjuvant and adjuvant therapy may confer more durable benefit than adjuvant therapy alone [38]. A meta-analysis by Akkus et al. (2026; 18 studies, 3478 patients) showed that ICI-based adjuvant therapy improved RFS (HR 0.51) and OS (HR 0.51) compared with surveillance, though most included studies were observational [47]. IMbrave050 (Qin 2023 [45]/Yopp 2026 [46]) and the adjuvant sintilimab trial (Wang K 2024 [34]) were not PVTT-specific studies; they enrolled high-risk HCC patients defined by microvascular invasion (MVI), multiple tumors, or large tumor size. Direct extrapolation of these results to patients with macrovascular PVTT requires caution, because CARES-009 included patients with vascular invasion (CNLC stage Ib–IIIa) but explicitly excluded Vp4 disease; therefore, its findings are applicable to Vp1–Vp3 but not to main portal trunk involvement. Dedicated prospective trials evaluating ICI-based adjuvant strategies specifically in the PVTT population remain an unmet need. Thus, results from ongoing phase 3 trials (CheckMate-9DX, EMERALD-2 etc.) are awaited.

## 7. Treatment Algorithm for Hepatocellular Carcinoma with Portal Vein Tumor Thrombus: An Evidence-Based Approach

Taken together, the evidence discussed above suggests that treatment selection for HCC with PVTT should be stratified by Vp classification (Vp1–Vp4), liver function (Child-Pugh class), and performance status (ECOG PS). For Vp1–Vp2 disease, surgical resection remains the first-line treatment in Child-Pugh A patients with PS 0–1, with adjuvant treatment considered for high-risk features. Borderline resectable or unresectable cases should receive systemic ICI-based therapy ± locoregional treatment (LRT), with conversion surgery considered upon achieving a response. For Vp3 disease, resection might be considered only in highly selected patients (Child-Pugh A, PS 0–1, AFP < 400 ng/mL, no portal hypertension, tumor confined to hemiliver), with adjuvant therapy considered on an individualized basis. Unresectable Vp3 patients should receive systemic therapy (atezolizumab + bevacizumab or durvalumab + tremelimumab) ± HAIC/TACE/radiotherapy (regionally adopted option), followed by conversion surgery assessment. For Vp4 disease, systemic ICI-based therapy ± HAIC ± radiotherapy (regionally adopted option) to PVTT is the standard approach, with conversion surgery reserved for responders meeting strict eligibility criteria: PVTT regression (Vp downstaging), Child-Pugh A, PS 0–1, adequate future liver remnant (≥40% with cirrhosis), no new extrahepatic disease, significant AFP decline, R0 resection feasibility, and multidisciplinary team consensus. Conversion surgery eligibility requires objective response (CR/PR by mRECIST), tumor confined to one lobe, absence of contralateral portal vein invasion, IVC tumor thrombus not extending to the atrium, and favorable tumor marker kinetics (AFP > 50% decline or PIVKA-II > 75% decline). The optimal postoperative treatment strategy after successful conversion surgery remains unclear. Some retrospective evidence suggests that TKI plus ICI therapy for 4–6 months may be feasible; however, further prospective validation is required. Key prognostic factors influencing treatment selection include AFP ≥ 400 ng/mL, tumor size > 5–7 cm, bilobar involvement, elevated bilirubin, portal hypertension, infiltrative growth pattern, and high ADV score (AFP × DCP × tumor volume). Patients with Child-Pugh B (Score ≥ 8), Child-Pugh C, or PS ≥ 2 may have to receive best supportive care regardless of Vp classification.

**Figure 2:**
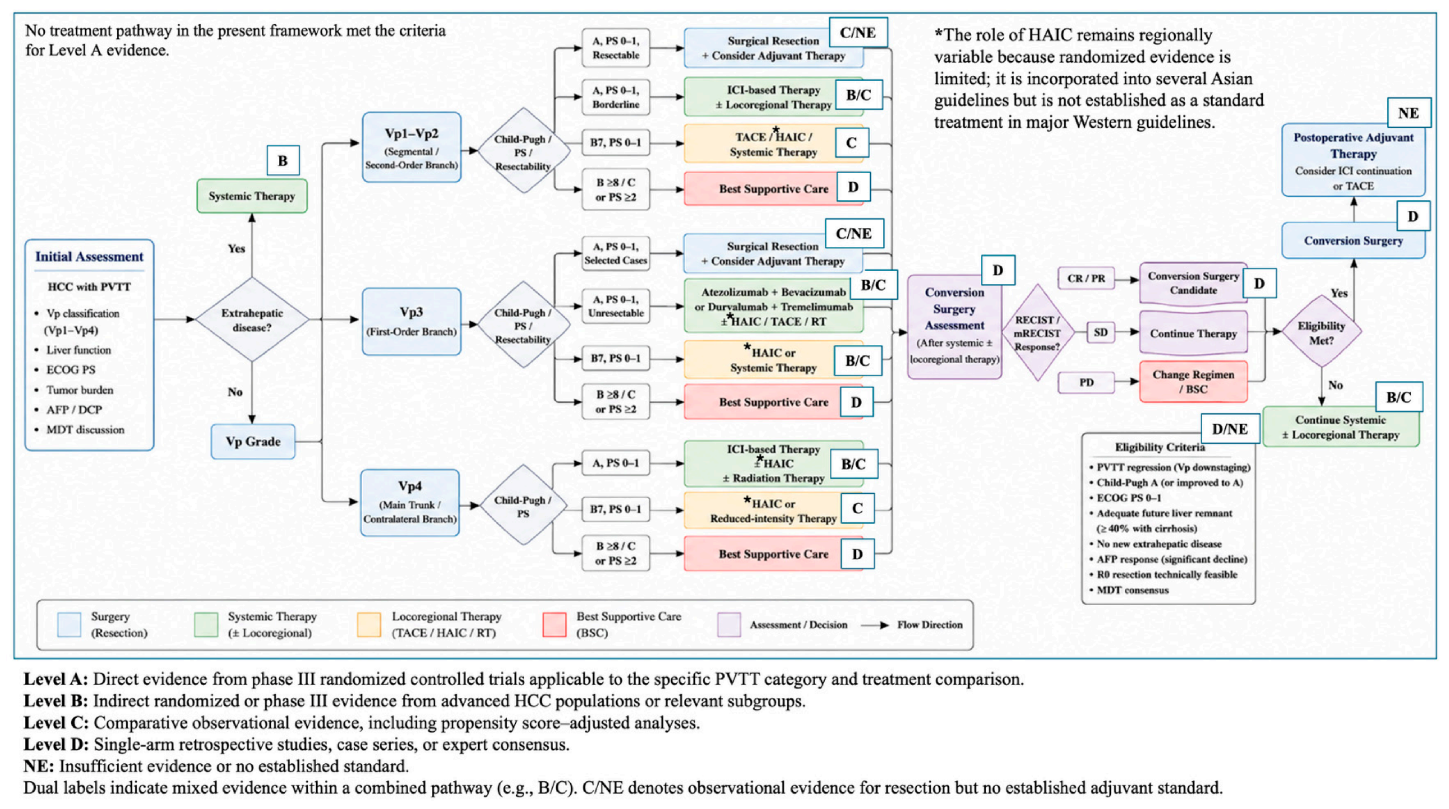
Proposed Multidisciplinary Decision framework for Hepatocellular Carcinoma With Portal Vein Tumor Thrombus According to Vp Classification and Liver Function Treatment strategies are stratified according to the extent of portal vein tumor thrombus (Vp1–Vp4), liver functional reserve, and performance status. The algorithm integrates surgical resection, systemic therapy, locoregional therapy, and conversion surgery in the modern immunotherapy era. HAIC, hepatic arterial infusion chemotherapy; ICI, immune checkpoint inhibitor; MDT, multidisciplinary team; PVTT, portal vein tumor thrombus; TACE, transarterial chemoembolization.

This algorithm, as shown in Figure 2, emphasizes the evolving role of systemic immunotherapy-based regimens and the potential for conversion surgery in carefully selected patients, while acknowledging that macrovascular invasion remains the most impactful adverse prognostic factor after hepatectomy. Although several studies have reported favorable outcomes of surgical resection and conversion surgery for HCC with PVTT, the level of evidence differs substantially among studies. Most data supporting surgery and conversion surgery are derived from retrospective cohort studies and meta-analyses including retrospective studies, which are inherently affected by patient selection bias. Therefore, these findings should be interpreted cautiously, and prospective randomized controlled trials are required to establish the true survival benefit and optimal treatment strategy.

## 8. East–West Differences in Treatment Strategies and Guideline Recommendations

Treatment recommendations for HCC with PVTT differ substantially across regions, particularly regarding the position of hepatic resection and locoregional therapy. As summarized in Table 5, major Western guidelines generally classify macrovascular invasion as advanced-stage disease and prioritize immune checkpoint inhibitor-based systemic therapy. The AASLD [74] and EASL guidelines [75] do not regard resection as standard treatment, although individualized surgery may be considered in highly selected patients after multidisciplinary discussion. ESMO [76] does not recommend surgery as a standard option, whereas ASCO focuses primarily on the selection of systemic therapy for advanced or unresectable HCC and does not position resection as a primary treatment strategy. Thus, the dominant Western approach remains systemic therapy, with surgery reserved, if considered at all, for exceptional cases.

Nevertheless, Western recommendations are not entirely uniform. The NCCN guidelines acknowledge that surgery may be considered in selected patients with major vascular invasion, although its role remains controversial, and explicitly incorporate the possibility of conversion surgery after a favorable response to systemic therapy. This position suggests an emerging shift from a strictly stage-based framework toward individualized multidisciplinary decision-making, although supporting evidence remains limited and resection is not established as the standard of care for Vp3/Vp4 disease.

Clinical outcomes reported from Asian cohorts have supported this more aggressive approach in selected patients with preserved liver function and locoregionally controllable disease. Surgical resection, HAIC, and TACE combined with radiotherapy have been associated with prolonged survival in selected populations [12,21,50,71]. However, these findings are derived predominantly from retrospective studies conducted at experienced centers. Although randomized evidence suggests that HAIC combined with sorafenib may improve outcomes in patients with major PVTT [12], randomized data from Western countries remain limited, and much of the available evidence is derived from retrospective East Asian cohorts. Consequently, no global consensus has been established regarding the role of HAIC. Several Asian guidelines include HAIC as an option for selected patients with advanced PVTT, whereas major Western guidelines prioritize systemic therapy and do not regard HAIC as a standard treatment. HAIC should therefore be considered a regionally adopted option for carefully selected patients at experienced centers rather than a universally established standard.

Western data remain comparatively limited; a retrospective study presented at ASCO GI 2023 reported a median overall survival of 7.2 months among 136 patients with PVTT, with generally poorer outcomes than those reported in selected Asian surgical cohorts [18]. Meta-analyses have also suggested that the benefit of resection is strongly dependent on PVTT extent: resection appears superior to TACE for Cheng type I and II disease, whereas a clear advantage has not been established for type III PVTT involving the main portal vein [51,52]. Beyond these region-specific approaches, TARE represents an important locoregional treatment option for selected patients with unresectable HCC and PVTT. In a systematic review and meta-analysis of 21 studies, Rognoni et al. reported pooled 1- and 3-year overall survival rates of 37% and 13%, respectively, in patients with advanced HCC due to portal vein thrombosis and preserved liver function, supporting the potential clinical benefit of TARE in this population [53].

Overall, the principal East–West difference lies not in the complete acceptance or rejection of surgery, but in the default treatment strategy and the threshold for considering resection. Western guidelines generally begin with systemic therapy and regard surgery as nonstandard or exceptional, whereas Asian guidelines more frequently incorporate resection and locoregional therapy into a resectability-based multidisciplinary framework. Importantly, even among Asian guidelines, surgery is not uniformly recommended for all patients with Vp3/Vp4 PVTT. The decision should therefore be individualized according to PVTT extent, hepatic reserve, tumor burden, extrahepatic disease, response to initial therapy, institutional expertise, and patient preferences. Given the absence of direct phase III comparisons between surgery and contemporary immunotherapy-based regimens, these regional differences should be interpreted as variations in clinical practice and evidence appraisal rather than proof of the superiority of one strategy over another.

## 9. Limitations, Evidence Gaps, and Future Perspectives

The evidence supporting hepatic resection for HCC with PVTT is derived predominantly from retrospective, nonrandomized studies and is therefore subject to selection bias and unmeasured confounding. Accordingly, the survival outcomes reported in individual studies should be interpreted as reflecting not only the effect of surgery itself, but also differences in patient selection, institutional expertise, treatment era, perioperative management, and treatment after recurrence. Substantial heterogeneity exists across studies in PVTT extent, tumor burden, liver function, portal hypertension, extrahepatic disease, and perioperative treatment. In a meta-analysis of 40 studies including 8218 patients, median overall survival differed markedly according to PVTT extent, ranging from 20.41 months in patients with segmental or second-order portal vein involvement to 6.41 months in those with main portal trunk invasion, with additional regional variation [60]. A nationwide study of 1590 patients also showed that long-term survival was associated not only with PVTT extent and tumor size, but also with liver function, cirrhosis, intraoperative blood loss, achievement of R0 resection, postoperative TACE, and treatment after recurrence [15]. Therefore, favorable outcomes reported in patients with branch-level PVTT and preserved liver function cannot be directly extrapolated to patients with VP3/VP4 disease. Moreover, surgery for VP3/VP4 PVTT may require major hepatectomy, tumor thrombectomy, or portal vein resection and reconstruction. Outcomes reported by highly specialized centers may therefore not be reproducible in lower-volume institutions [13]. Earlier studies commonly used sorafenib as the principal nonsurgical comparator [10], whereas contemporary treatment includes immune checkpoint inhibitor-based combinations, molecular-targeted agents, HAIC, radiotherapy, and other multimodal strategies. The reported improvement in overall survival in a later treatment era, despite no clear improvement in recurrence-free survival, suggests that advances in perioperative care and post-recurrence treatment also influence prognosis [54].

Important evidence gaps remain. No phase III randomized trial has directly compared hepatic resection with contemporary immunotherapy-based systemic treatment in patients with VP3 or VP4 disease. In addition, treatment regimens, response criteria, definitions of resectability, and the timing of conversion surgery have not been standardized, and patients who proceed to surgery after a favorable response are subject to treatment-response selection bias [61]. Biological resectability also remains an exploratory concept, and neither a validated model for predicting a meaningful survival benefit from surgery nor an established postoperative adjuvant strategy is currently available.

Future studies should standardize PVTT classification, liver function assessment, conversion-surgery criteria, and outcome definitions, and should compare surgery with contemporary systemic and multimodal therapies in prospective international multicenter settings. Selected ongoing randomized trials relevant to HCC with major PVTT or macrovascular invasion are summarized in Table 6. These trials are expected to provide important evidence regarding the integration of immunotherapy, HAIC, radiotherapy, and other locoregional treatments in patients with advanced PVTT. However, only a limited number of ongoing trials specifically focus on Vp3/Vp4 PVTT, highlighting the continued need for adequately powered randomized studies in this population. Until such evidence becomes available, surgery and conversion surgery for advanced PVTT should be regarded as conditional options for highly selected patients rather than established standards of care.

## Figures and Tables

**Table 1:** Selected Studies Evaluating Prognostic Factors in Hepatocellular Carcinoma With Portal Vein Tumor Thrombosis.

Author	Year	Study Design	No. of Patients (Eligibility)	Comparison	Survival Outcomes	Poor Prognostic Factors (Multivariate Analysis)
Roayaie et al. [56]	2013	Retrospective cohort (single-center)	*n* = 165; HCC with macroscopic vascular invasion (hepatic vein and/or portal vein) undergoing hepatectomy (Mount Sinai, USA)	Prognostic factors after resection of HCC with macroscopic vascular invasion	MST 13.1 months; 5-year OS 14%; perioperative mortality 7.3%	AFP > 30 ng/mL (HR 2.07), tumor size > 7 cm (HR 1.59), extent of vascular invasion (HR 1.74)
Faber et al. [57]	2014	Retrospective cohort (single-center)	*n* = 107; HCC with vascular invasion (micro- and macrovascular) undergoing hepatectomy (Germany)	Microvascular vs. macrovascular invasion	1-/3-/5-year OS: 64.3%/41.4%/23.9%; MST 19 months	Lymphatic invasion, intraoperative transfusion, FFP use, prolonged ICU stay, elevated preoperative bilirubin
Kokudo et al. [21]	2016	Nationwide retrospective cohort	*n* = 6474; HCC with Vp1–Vp4 PVTT (resection *n* = 2093 vs. non-resection *n* = 4381); Japanese nationwide survey	Resection vs. non-resection	MST: resection 2.87 years vs. non-resection 1.10 years (*p* = 0.001); PSM: 2.45 vs. 1.57 years; 90-day mortality 3.7%	Vp4 (main trunk/contralateral branch invasion): survival benefit of resection was lost
Huo et al. [16]	2019	Retrospective cohort (single-center)	*n* = 487; HCC with Vp1–Vp4 PVTT undergoing hepatectomy (EHBH, China)	Factors for early death (≤3 months) vs. long-term survival (≥2 years)	(Factor analysis of short- and long-term survival)	Early death: elevated AFP, elevated T-Bil, radiologic ascites; Long-term poor prognosis: elevated AFP, clinically significant portal hypertension, extensive PVTT, poor differentiation
Zhang et al. [19]	2019	Multicenter retrospective cohort (development/validation study)	*n* = 1327 (training *n* = 432 + validation *n* = 895); HCC with Vp1–Vp4 PVTT undergoing R0 hepatectomy (multicenter, China)	Prognostic stratification by EHBH-PVTT score	Score ≤ 3: MST 17.0 months vs. >3: MST 7.9 months (*p* < 0.001)	Elevated T-Bil, elevated AFP, large tumor size, presence of satellite nodules (score components)
Chen et al. [15]	2020	Nationwide retrospective cohort	*n* = 1590; HCC with Vp1–Vp4 PVTT undergoing hepatectomy (Chinese nationwide database)	Actual 3-year survivors vs. non-survivors	3-year actuarial survival 16.6%; 3-year actual survival 11.7%	T-Bil > 17.1 μmol/L, AFP > 400 ng/mL, extensive PVTT, tumor size > 5 cm, intraoperative blood loss > 400 mL, non-R0 resection, liver cirrhosis, absence of tumor capsule, early recurrence (≤1 year)
Hwang et al. [20]	2021	Retrospective cohort (single-center)	*n* = 147; HCC with Vp1–Vp4 PVTT undergoing hepatectomy (Asan Medical Center, Korea)	Prognostic prediction by ADV score	5-year recurrence rate 79.2%; 5-year HCC-specific survival 43.5%	High ADV score (AFP × DCP × tumor volume); Vp classification was not a significant prognostic factor
Mähringer-Kunz et al. [2]	2021	Retrospective cohort (single-center)	*n* = 278; HCC with Vp1–Vp4 PVTT; all treatment modalities (resection, TACE/SIRT, sorafenib, BSC) (single center, Germany)	OS by Vp classification and treatment modality	Resection group OS: Vp1 = 32.4 months, Vp2 = 10.7 months, Vp3 = 6.6 months, Vp4 = 8.0 months	Advanced PVTT extent (Vp3–Vp4)
Komatsu et al. [3]	2022	Retrospective cohort (single-center)	*n* = 100; HCC with Vp3 (*n* = 37) or Vp4 (*n* = 63) PVTT undergoing hepatectomy (Kobe University, Japan)	Vp3 vs. Vp4; R0/R1 vs. R2 resection	MST 14.5 months; 1-year OS 59.6%, 3-year OS 16.8%; Vp3 vs. Vp4: no significant difference (*p* = 0.71)	None of the conventional factors (Vp classification, resection completeness, tumor size, tumor number) reached significance (tumor biology dominant)
Kim et al. [14]	2023	Retrospective cohort (single-center)	*n* = 161; HCC with Vp1–Vp4 PVTT undergoing hepatectomy (Asan Medical Center, Korea)	Vp1–Vp2 vs. Vp3–Vp4; prognostic factor analysis	1-/3-/5-year OS: 65.0%/38.4%/36.0%; 1-year RFS: 25.5%	Esophageal varices, tumor size > 5 cm, bilobar involvement, non-anatomical resection, non-R0 resection; RFS: presence of MVI
Zhang et al. [17]	2023	Retrospective cohort (single-center)	*n* = 94; HCC with Vp1–Vp4 PVTT undergoing hepatectomy (China)	MRI-based prognostic prediction model	MST 15.4 months; high-risk 11.7 months vs. low-risk 25.0 months	Sum of two largest tumor diameters (HR 3.05), infiltrative growth pattern (HR 1.93)
ASCO GI 2023 [18]	2023	Conference abstract (retrospective cohort)	*n* = 136; HCC with Vp1–Vp4 PVTT; all treatment modalities (single US center)	OS by Vp classification and treatment modality	MST 7.2 months; Child-Pugh A/B/C: 16.0/3.6/1.9 months	Child-Pugh B/C, Vp4 (4.7 vs. 12.8 months, *p* = 0.055)
Xu et al. [22]	2024	Retrospective cohort (single-center)	*n* = 362; HCC with Vp1–Vp4 PVTT undergoing hepatectomy (China)	MVI-positive vs. MVI-negative after hepatectomy	MVI-negative: mOS 27.1 months vs. MVI-positive: 13.7 months (*p* = 0.014)	Presence of MVI: OS (HR 1.482; *p* = 0.045), RFS (HR 1.601; *p* = 0.009)
Yao et al. [23]	2025	Retrospective cohort (30-year single-center study)	*n* = 3981 (PVTT surgery *n* = 156, PVTT non-surgery *n* = 1842, non-PVTT surgery *n* = 1983); HCC ± PVTT (Queen Mary Hospital, Hong Kong, 30 years)	PVTT surgery vs. PVTT non-surgery vs. non-PVTT surgery	PVTT surgery: mOS 13.0 months; PVTT non-surgery: mOS 2.7 months	AFP ≥ 17,400 ng/mL (mOS 8.2 vs. 16.2 months, *p* < 0.001); PVTT itself as a strong adverse prognostic factor

AFP, alpha-fetoprotein; ADV score, AFP × DCP × tumor volume score; BSC, best supportive care; DCP, des-gamma-carboxy prothrombin; EHBH, Eastern Hepatobiliary Surgery Hospital; FFP, fresh frozen plasma; HCC, hepatocellular carcinoma; HR, hazard ratio; ICU, intensive care unit; mOS, median overall survival; MRI, magnetic resonance imaging; MST, median survival time; MVI, microvascular invasion; OS, overall survival; PSM, propensity score matching; PVTT, portal vein tumor thrombus; RFS, recurrence-free survival; R0, microscopically margin-negative resection; R1, microscopically margin-positive resection; R2, macroscopically residual tumor; SIRT, selective internal radiation therapy; TACE, transarterial chemoembolization; T-Bil, total bilirubin; Vp1, tumor thrombus in the third-order or more peripheral portal vein branches; Vp2, tumor thrombus in the second-order portal vein branches; Vp3, tumor thrombus in the first-order portal vein branches; Vp4, tumor thrombus in the main trunk or contralateral branch of the portal vein. Vp classification is based on the General Rules for the Clinical and Pathological Study of Primary Liver Cancer by the Liver Cancer Study Group of Japan.

**Table 2:** Overall survival outcomes according to the extent of portal vein tumor thrombus (VP classification) in hepatocellular carcinoma according to the Report of the 23rd nationwide follow-up survey of primary liver cancer in Japan (2014–2015). Survival is expressed as median survival time (MST) and 1-, 3-, and 5-year overall survival (OS) rates. Abbreviations: VP, portal vein invasion; MST, median survival time; OS, overall survival.

VP Classification	MST (Months)	1-Year OS	3-Year OS	5-Year OS
Vp0	106.97	94.3%	83.4%	72.9%
Vp1	80.56	85.2%	66.5%	56.9%
Vp2	36.96	71.1%	50.4%	42.9%
Vp3	24.84	64.2%	42.0%	33.1%
Vp4	14.09	54.4%	23.3%	19.2%

**Table 3:** Summary of Studies on Neoadjuvant, Conversion, and Perioperative Therapies for Hepatocellular Carcinoma With Portal Vein Tumor Thrombus.

Author	Year	Study Design	No. of Patients (Eligibility)	Comparison	Survival Outcomes	Poor Prognostic Factors (Multivariate Analysis)
Wei et al. [30]	2019	Phase III randomized controlled trial (RCT)	*n* = 164; resectable HCC with PVTT	Neoadjuvant 3D-CRT + resection vs. resection alone	24-month OS: 27.4% vs. 9.4% (*p* < 0.001)	High IL-6 expression (radiation resistance predictor)
Hu et al. [31]	2023	Retrospective cohort (single-center)	*n* = 120; HCC with PVTT (neoadjuvant HAIC + resection vs. resection alone)	Neoadjuvant HAIC + resection vs. resection alone	5-year OS: 66.4% vs. 37.2% (*p* < 0.001); benefit confined to HAIC responders	Non-response to HAIC; AFP and CRP predictive of response (AUC 0.756)
Yuan et al. [37]	2023	Multicenter retrospective cohort	Multicenter cohort; HCC with PVTT after resection	TACE + ICI vs. TACE alone (postoperative adjuvant)	Improved DFS and OS with TACE + ICI (specific values per original study)	—
D’Alessio et al. (NeoHCC Consortium) [66]	2024	Cross-trial pooled analysis (patient-level analysis)	*n* = 111; HCC after neoadjuvant ICI (cross-trial pooled analysis)	MPR vs. non-MPR after neoadjuvant ICI	MPR: mRFS not reached vs. 28.3 months (HR 0.26; *p* = 0.0024); pCR rate 18%, MPR rate 32%	Non-MPR; 30% of MPR patients had no radiological response (RECIST discordance)
Wang et al. [34]	2024	Retrospective cohort (single-center)	*n* = 93; initially unresectable HCC with PVTT after successful conversion (LRT + TKI + anti-PD-1)	Surgical vs. non-surgical group after successful conversion	No significant OS or PFS difference; CR and Cheng type III–IV tended to fare better without surgery	—
Xu et al. (SR/MA) [32]	2024	Systematic review and meta-analysis	*n* = 4042 (38 studies); initially unresectable HCC	Conversion surgery vs. continued systemic therapy; conversion rates by regimen	Conversion surgery: OS HR 0.45, PFS HR 0.49; conversion rate: TKI alone 8%, TKI + anti-PD-1 28%, TKI + anti-PD-1 + LRT 33–35%	—
Wang et al. (CARES-009, RCT) [38]	2025	Phase II/III randomized controlled trial (RCT)	*n* = 294; resectable HCC at intermediate/high recurrence risk (CNLC Ib–IIIa, Vp4 excluded)	Perioperative camrelizumab + rivoceranib vs. surgery alone	Significantly improved EFS; increased MPR rate; consistent benefit in vascular invasion subgroup	≥90% tumor necrosis as optimal threshold for EFS benefit
Rajagopal et al. (SR/MA) [29]	2026	Systematic review and meta-analysis	*n* = 621 (7 trials); HCC with PVTT	Neoadjuvant therapy + resection vs. resection alone	OS: HR 0.48 (*p* < 0.001); RFS: HR 0.40 (*p* < 0.001); I^2^ = 0.00	—
Zhang et al. (Phase 2) [33]	2026	Phase II prospective trial	*n* = 142; unresectable intermediate-advanced HCC	LEN-TAP (lenvatinib + TACE + anti-PD-1) vs. TACE alone	Conversion rate: 59.2% vs. 18.3% (*p* < 0.001); ORR: 78.9% vs. 16.9%; mOS: not reached vs. 23.0 months (*p* < 0.001)	—
Wang et al. [35]	2026	Retrospective cohort (multicenter)	*n* = 124; initially unresectable HCC with PVTT after R0 conversion	Optimal adjuvant duration: 4–6 months vs. ≥7 months of TKI + ICI	No significant OS or PFS difference between 4–6 months and ≥7 months (plateau effect); 4–6 months recommended	—

Abbreviations: HCC, hepatocellular carcinoma; PVTT, portal vein tumor thrombus; 3D-CRT, three-dimensional conformal radiotherapy; HAIC, hepatic arterial infusion chemotherapy; TACE, transarterial chemoembolization; ICI, immune checkpoint inhibitor; TKI, tyrosine kinase inhibitor; LRT, locoregional therapy; LEN-TAP, lenvatinib plus TACE plus anti-PD-1; OS, overall survival; RFS, recurrence-free survival; PFS, progression-free survival; EFS, event-free survival; DFS, disease-free survival; mOS, median overall survival; mRFS, median recurrence-free survival; HR, hazard ratio; ORR, objective response rate; MPR, major pathological response; pCR, pathological complete response; CR, complete response; AFP, alpha-fetoprotein; CRP, C-reactive protein; IL-6, interleukin-6; RECIST, Response Evaluation Criteria in Solid Tumors; RCT, randomized controlled trial; SR, systematic review; MA, meta-analysis; CNLC, China Liver Cancer staging; R0, margin-negative resection.

**Table 4:** Summary of Studies on Adjuvant Therapy After Surgical Resection for Hepatocellular Carcinoma With or At Risk of Portal Vein Tumor Thrombus.

Author	Year	Study Design	No. of Patients (Eligibility)	Comparison	Survival Outcomes	Poor Prognostic Factors
Sun H et al. [44]	2023	Systematic review and Bayesian network meta-analysis	*n* = 1927 (14 trials); HCC with PVTT after resection	Adjuvant RT vs. TACE vs. HAIC vs. sorafenib vs. surgery alone	RT: OS HR 0.38, RFS HR 0.27 (best); all adjuvant therapies superior to surgery alone	Not reported (network meta-analysis)
Peng Z et al. [43].	2024	Multicenter randomized controlled trial (RCT)	*n* = 158; HCC with Cheng type I–III PVTT after R0 resection	Adjuvant TACE + sorafenib vs. sorafenib alone	TACE + SOR: mRFS 16.8 vs. 12.6 mo (HR 0.57; *p* = 0.002); mOS 30.4 vs. 22.5 mo (HR 0.57; *p* = 0.017)	Age 50, male sex, multiple tumors, tumor ≥ 5 cm, AFP 400 ng/mL favored combination (subgroup analysis)
Qin S et al. [45] (IMbrave050)	2023	Phase III randomized controlled trial (RCT)	*n* = 668; high-risk HCC (including MVI/vascular invasion) after resection or ablation (global, 26 countries)	Adjuvant atezo + bev vs. active surveillance	1st IA (f/u 17.4 mo): RFS HR 0.72 (95% CI 0.53–0.98; *p* = 0.012)	Not reported
Yopp A et al. (IMbrave050 updated) [46]	2026	Updated analysis of Phase III randomized controlled trial	*n* = 668; same population as above	Adjuvant atezo + bev vs. active surveillance	2nd IA (f/u 35.1 mo): RFS HR 0.90 (95% CI 0.72–1.12); OS HR 1.26 (95% CI 0.85–1.87)—benefit not sustained	Not reported
Taddei TH et al. (AASLD Critical Update [73])	2025	Practice guideline/Critical update	N/A (guideline)	Adjuvant/neoadjuvant systemic therapy vs. no adjuvant therapy	AASLD advises against adjuvant or neoadjuvant systemic therapies after resection based on IMbrave050 updated data	N/A
Wang Z et al. (CARES-009 [38])	2025	Phase II/III randomized controlled trial (RCT)	*n* = 294; resectable HCC, CNLC Ib–IIIa (including vascular invasion, excluding Vp4), China	Perioperative camrelizumab + rivoceranib vs. surgery alone	mEFS 42.1 vs. 19.4 mo (HR 0.59; *p* = 0.004); Grade ≥ 3 TRAE 38% vs. 0%; 2 treatment-related deaths	CNLC stage used for stratification; details not fully reported
Wang K et al. [48]	2024	Phase II randomized controlled trial (RCT)	*n* = 198; HCC with MVI (not macrovascular PVTT) after resection, China	Adjuvant sintilimab vs. active surveillance	mRFS 27.7 vs. 15.5 mo (HR 0.534; *p* = 0.002); Grade 3–4 TRAE 12.4%	Not reported for multivariate; MVI as enrollment criterion
Akkus E et al. [47]	2026	Systematic review and meta-analysis	*n* = 3478 (18 studies); HCC after resection or ablation	Adjuvant ICI-based therapy vs. surveillance (meta-analysis)	RFS HR 0.51 (95% CI 0.44–0.60; *p* < 0.001); OS HR 0.51 (95% CI 0.40–0.65; *p* < 0.001)	Not applicable (meta-analysis); benefit consistent regardless of curative modality
Xu X et al. [49]	2023	Multicenter retrospective cohort (propensity score–matched analysis)	*n* = 627 (146 ICI, 481 no ICI); BCLC B/C HCC after resection, multicenter prospective database (China)	Adjuvant ICI vs. no adjuvant ICI (PSM: 99 pairs)	After PSM: mRFS 29.6 vs. 19.3 mo (*p* = 0.031); mOS 35.1 vs. 27.8 mo (*p* = 0.036)	Adjuvant ICI: independent factor for RFS (HR 0.630; *p* = 0.015) and OS (HR 0.601; *p* = 0.013)
Yuan L et al. [37]	2023	Multicenter retrospective cohort	*n* = 90 (42 TACE + ICI, 48 TACE alone); HCC with PVTT after R0 resection, multicenter (China)	Adjuvant TACE + ICI vs. adjuvant TACE alone	mRFS 12.76 vs. 8.11 mo (*p* = 0.038); mOS 24.5 vs. 19.1 mo (*p* = 0.032)	TACE + ICI: independent factor for RFS (HR 0.54; *p* = 0.019) and OS (HR 0.47; *p* = 0.014)

Abbreviations: AFP, alpha-fetoprotein; atezo, atezolizumab; bev, bevacizumab; BCLC, Barcelona Clinic Liver Cancer; CNLC, China Liver Cancer Staging; f/u, follow-up; HAIC, hepatic arterial infusion chemotherapy; HCC, hepatocellular carcinoma; HR, hazard ratio; IA, interim analysis; ICI, immune checkpoint inhibitor; mEFS, median event-free survival; mo, months; mOS, median overall survival; mRFS, median recurrence-free survival; MVI, microvascular invasion; OS, overall survival; PSM, propensity score matching; PVTT, portal vein tumor thrombus; RFS, recurrence-free survival; RT, radiotherapy; SOR, sorafenib; TACE, transarterial chemoembolization; TRAE, treatment-related adverse event.

**Table 5:** Detailed Recommendations of Major International Guidelines for the Management of Hepatocellular Carcinoma With Portal Vein Tumor Thrombosis.

Guideline/Region	Position of PVTT	Recommended Primary Treatment	Role of Surgical Resection	Key Characteristics
AASLD 2023 (USA)	Advanced HCC with macrovascular invasion	Systemic therapy (ICI-based regimens)	Not standard; may be considered in highly selected cases after MDT discussion	PVTT is regarded as a marker of advanced disease; systemic therapy is the mainstay, with increasing emphasis on multidisciplinary care
EASL 2021/2025 (Europe)	BCLC stage C (portal invasion)	Systemic therapy (immunotherapy-based)	Not standard; individualized decisions possible in selected cases	Maintains BCLC framework but increasingly emphasizes individualized and multidisciplinary approaches
ESMO 2024/2025 (Europe)	BCLC C (vascular invasion)	Atezolizumab + bevacizumab or other ICI-based regimens	Not standard	Oncology-driven guideline with clearly defined systemic treatment algorithms
ASCO 2024	Advanced HCC	Immune checkpoint inhibitor-based systemic therapy	Not addressed as a primary option	Focused on systemic therapy selection in unresectable/advanced disease
APASL 2024 (Asia-Pacific)	Macrovascular invasion as a major stratification factor	Combination of systemic and locoregional therapies (ICI + TACE/HAIC, etc.)	Considered in resectable cases	Explicit integration of systemic and locoregional therapies; evolving toward multimodal strategies
KLCA-NCC (Korea)	Vascular invasion included in mUICC staging	Surgery, TACE, or systemic therapy depending on stage and liver function	Considered in selected patients	mUICC-based; multimodal treatment widely used in clinical practice
Chinese PVTT Guideline 2021	PVTT as an independent prognostic and therapeutic stratification factor	Multidisciplinary treatment (surgery, TACE, RT, HAIC, systemic therapy)	Actively recommended, including after downstaging	PVTT-specific guideline; most aggressive treatment approach
JSH 2025 (Japan)	HCC with vascular invasion (including PVTT)	Resectable: surgical resection (strong recommendation) Unresectable: systemic therapy (strong recommendation) Others: HAIC, TACE, RT	First-line treatment in resectable cases	Resectability-based stratification; PVTT is not uniformly regarded as a systemic disease
NCCN v1.2026 (USA)	Major vascular invasion; controversial but potentially resectable	Systemic therapy (ICI-based regimens, Category 1); locoregional therapy for limited PV invasion	Controversial but can be considered for major vascular invasion; conversion surgery after systemic response explicitly mentioned	Unique among Western guidelines in explicitly listing major vascular invasion as a potential (though controversial) surgical indication; emphasizes MDT review; includes conversion surgery concept

AASLD: American Association for the Study of Liver Diseases; APASL: Asian Pacific Association for the Study of the Liver; ASCO: American Society of Clinical Oncology; BCLC: Barcelona Clinic Liver Cancer; EASL: European Association for the Study of the Liver; ESMO: European Society for Medical Oncology; HAIC: hepatic arterial infusion chemotherapy; HCC: hepatocellular carcinoma; ICI: immune checkpoint inhibitor; JSH: Japan Society of Hepatology; KLCA-NCC: Korean Liver Cancer Association–National Cancer Center; MDT: multidisciplinary team; mUICC: modified Union for International Cancer Control; PVTT: portal vein tumor thrombus; RT: radiotherapy TACE: transarterial chemoembolization.

**Table 6:** Selected ongoing randomized trials relevant to hepatocellular carcinoma with major portal vein tumor thrombosis or macrovascular invasion.

Trial; Phase; Status	Key Eligibility Criteria	Treatment Regimens	Primary Endpoint	Estimated Study Completion	Relevance to Vp3/Vp4 PVTT
**ALICE** (NCT06904170); phase II/III; recruiting	Unresectable HCC with a high tumor burden, defined as Vp4 PVTT, Vp3 PVTT with bilobar tumor involvement, or tumor involvement of >50% of the liver; Child–Pugh class A; ECOG PS 0–1; extrahepatic spread permitted	**Experimental:** durvalumab plus tremelimumab combined with HAIC using GEMOX (gemcitabine and oxaliplatin) **Control:** durvalumab plus tremelimumab alone	Phase II: ORR according to RECIST 1.1; Phase III: OS	September 2030	Directly includes patients with Vp4 PVTT and selected patients with Vp3 PVTT
**STRATUM/AHCC09** (NCT05377034); phase II; recruiting	Locally advanced HCC without extrahepatic metastases; tumor burden beyond the up-to-7 criteria and/or vascular invasion classified as Vp1–Vp3 or Vv1–Vv2	**Experimental:** SIRT with yttrium-90 resin microspheres followed by atezolizumab plus bevacizumab **Control:** SIRT with yttrium-90 resin microspheres followed by intravenous placebo	Best overall response rate at 9 months after randomization, assessed according to RECIST 1.1 and mRECIST	January 2028	Includes Vp3 PVTT but excludes Vp4 PVTT
**HELIO-RT/NRG-GI012** (NCT07166406); phase III; recruiting	Advanced HCC with radiologically confirmed macrovascular invasion; Child–Pugh class A or B7; ECOG PS 0–2; extrahepatic disease permitted; no previous systemic therapy or TARE	**Experimental:** investigator-selected IO-based systemic therapy plus liver-directed SBRT **Control:** the same IO-based systemic therapy without SBRT	OS	March 2029	Includes patients with macrovascular invasion, but eligibility is not defined according to the Japanese Vp classification

**Abbreviations:** ECOG PS, Eastern Cooperative Oncology Group performance status; GEMOX, gemcitabine plus oxaliplatin; HAIC, hepatic arterial infusion chemotherapy; HCC, hepatocellular carcinoma; IO, immunotherapy; mRECIST, modified Response Evaluation Criteria in Solid Tumors; ORR, objective response rate; OS, overall survival; PVTT, portal vein tumor thrombosis; RECIST, Response Evaluation Criteria in Solid Tumors; SBRT, stereotactic body radiation therapy; SIRT, selective internal radiation therapy; TARE, transarterial radioembolization. **Note:** Recruitment status and estimated completion dates were obtained from the respective clinical trial registries and verified on 16 July 2026. Registry-reported completion dates are subject to change. Because the Japanese Vp classification was not used in all trials, the direct applicability of each trial to patients with Vp3/Vp4 PVTT is indicated separately.

## Data Availability

No new data were created or analyzed in this study. Data sharing is not applicable to this article.

## Abbreviations

AASLD	American Association for the Study of Liver Diseases
ADV	alpha-fetoprotein × des-gamma-carboxy prothrombin × tumor volume
AFP	alpha-fetoprotein
ALBI	albumin-bilirubin
APASL	Asian Pacific Association for the Study of the Liver
ASCO	American Society of Clinical Oncology
BCLC	Barcelona Clinic Liver Cancer
BR	borderline resectable
BSC	best supportive care
CARES	Camrelizumab and Rivoceranib Evaluation Study
CNLC	China Liver Cancer staging
CR	complete response
CRP	C-reactive protein
CT	computed tomography
DCP	des-gamma-carboxy prothrombin
DFS	disease-free survival
DSS	disease-specific survival
EASL	European Association for the Study of the Liver
ECOG PS	Eastern Cooperative Oncology Group performance status
EFS	event-free survival
ESMO	European Society for Medical Oncology
HAIC	hepatic arterial infusion chemotherapy
HBP	hepato-biliary-pancreatic
HCC	hepatocellular carcinoma
HR	hazard ratio
IA	interim analysis
ICI	immune checkpoint inhibitor
ICG-R15	indocyanine green retention rate at 15 min
IL-6	interleukin-6
IVC	inferior vena cava
JSH	Japan Society of Hepatology
JSHBPS	Japanese Society of Hepato-Biliary-Pancreatic Surgery
KLCA-NCC	Korean Liver Cancer Association–National Cancer Center
LEN-TAP	lenvatinib plus transarterial chemoembolization plus anti-PD-1 therapy
LRT	locoregional therapy
MDT	multidisciplinary team
mEFS	median event-free survival
MPR	major pathological response
MRI	magnetic resonance imaging
mRECIST	modified Response Evaluation Criteria in Solid Tumors
MST	median survival time
MVI	microvascular invasion
NCCN	National Comprehensive Cancer Network
ORR	objective response rate
OS	overall survival
pCR	pathological complete response
PFS	progression-free survival
PR	partial response
PSM	propensity score matching
PVTT	portal vein tumor thrombus
RCT	randomized controlled trial
RECIST	Response Evaluation Criteria in Solid Tumors
RFS	recurrence-free survival
RT	radiotherapy
SOR	sorafenib
TACE	transarterial chemoembolization
TKI	tyrosine kinase inhibitor
TRAE	treatment-related adverse event
Vp	portal vein tumor thrombus classification
